# Analysis of molecular marker expression in cutaneous lesions and cervical carcinoma associated with HPV infection

**DOI:** 10.25122/jml-2023-0329

**Published:** 2024-06

**Authors:** Laura Maghiar, Liliana Sachelarie, Anca Carmen Huniadi

**Affiliations:** 1 Department of Dental Medicine, Faculty of Medicine and Pharmacy, University of Oradea, Oradea, Romania; 2 Department of Preclinical Discipline, Apollonia University, Iasi, Romania

**Keywords:** HPV infection, molecular markers, malignant transformation

## Abstract

The study sought to systematically compare the expression of molecular markers in benign cutaneous lesions and squamous cell cervical carcinoma associated with HPV infection to better understand the pathophysiological mechanisms involved in HPV-related lesions and their progression to malignancy. We included 200 patients recruited from a gynecological clinic divided into two groups: 100 patients with positive HPV tests presenting with cutaneous lesions and 100 patients diagnosed with squamous cell cervical carcinoma and testing positive for HPV. The participants were selected to ensure diverse ethnic and demographic representation. The study utilized different statistical analyses, including Chi-square tests to assess associations between categorical variables and logistic regression to evaluate factors influencing lesion progression and compare marker expressions across different lesion types. The results indicated significant differences in the expression of specific molecular markers between cutaneous lesions and cervical carcinomas, highlighting distinct molecular pathways involved in HPV-related lesion development. Notably, markers such as p16, p53, and E-cadherin showed varying expression, suggesting their potential role in distinguishing between benign and malignant lesions. The findings emphasize the significance of molecular marker profiling in improving diagnostic and therapeutic strategies for HPV-related lesions. The differential expression of molecular markers can offer valuable insights into the pathogenesis of HPV-induced lesions and help develop targeted interventions to prevent malignant transformation. Further research is necessary to validate these markers in larger cohorts and diverse populations.

## INTRODUCTION

Human Papillomavirus (HPV) infection is one of the most common viral infections transmitted through sexual contact, significantly impacting public health due to its association with a wide range of lesions. These include benign lesions, such as papillomas and skin warts, as well as premalignant and malignant lesions, particularly on the cervix and skin. In recent years, studies have focused very intensively on understanding the mechanisms by which HPV infection contributes to the development of cervical cancer but also to the development of other neoplasias, highlighting the importance of studying skin lesions in association with this virus [1,2].

HPV is classified into different genotypes based on their oncogenic potential. Low-risk HPV genotypes (LR-HPV), including HPV 6, 11, 27, 32, 42, 53, 54, 57, 61, 62, 69, 71, 72, 81, 83, 84, 86, 87, 89, 90, 102, and 106, are primarily responsible for causing cutaneous, external genital, and perianal warts, with HPV 6 and 11 accounting for approximately 90% of these warts. Conversely, 12 high-risk HPV genotypes (HR-HPV), including HPV 16, 18, 31, 33, 35, 39, 45, 51, 52, 56, 58, 59, and 66, are associated with the development of precancerous lesions and are implicated in 98% of cervical cancers, as well as vulvar, penile, anal, and oropharyngeal cancers. Notably, HPV 16 and 18 are responsible for 70% of precancerous lesions and cervical cancers. In addition, HR-HPVs are also occasionally detected in external and perianal warts, usually together with LR-HPV genotypes. Another subset of HPV genotypes, including 26, 30, 34, 53, 67, 68, 69, 70, 73, 82, 85, and 97, is classified as having uncertain risk, with their oncogenic potential still under investigation [3,4].

Effective diagnosis and management of HPV-induced skin and cervical lesions require close collaboration between dermatologists and gynecologists. HPV-induced premalignant and malignant genital lesions can manifest both on the skin and on the vaginal and cervical mucosa. Benign HPV-induced skin lesions are primarily represented by warts, hyperkeratotic nodules most commonly found on the lower and upper limbs, but they can appear anywhere on glabrous skin. Morphology can vary considerably from relatively smooth, sessile lesions to large pedunculated lesions. Condylomata acuminata are tumoral lesions of different sizes with a conopidiform appearance. These lesions are whitish, soft, and multifocal, with a tendency to group into plaques, with HPV types 6 and 11 frequently identified in these lesions.

Bowen's disease is a precancerous dyskeratosis on the skin or mucous membranes, which is clinically characterized by erythematous, scaly, or crusted plaques. The malignancy potential of Bowen's disease ranges between 30% and 50%, particularly when it occurs in the perineal region, where it may be associated with condyloma acuminatum. Additionally, when Bowen's disease is found in areas not exposed to the sun, it may be related to HPV infection [5,6].

The two-tier system of low-grade SIL (LSIL) and high-grade SIL (HSIL) matches the carcinogenic potential of HPV and allows better communication between pathologists. LSIL refers to changes associated with transient HPV infection, while HSIL identifies true precancerous lesions. However, depending on qualitative and quantitative factors, some equivocal morphological features may fall into the category of 'Atypical Squamous Cells' (ASC), which are subdivided into two categories; 'atypical squamous cells of undetermined significance' (ASC-US) or 'atypical squamous cells-HSIL cannot be excluded' (ASC-H), based on the underlying suspected LSIL versus HSIL lesion, respectively. HSIL refers to morphologic changes associated with the upper end of the SIL spectrum and includes both CIN 2 and CIN 3 (with CIS). HSIL has a higher rate of progression to cancer and a lower rate of regression. Long-term progression to invasive cancer is estimated at 30% over 30 years. HSIL can be found with any other squamous or glandular abnormalities [7,8].

Globally, cervical cancer ranks as the fourth most common cancer among women, with around 570,000 new cases reported annually, approximately 85% of which occur in less developed regions. In 2018, cervical cancer was responsible for about 311,000 deaths, representing 7.5% of all cancer-related deaths among women [9].

At the molecular level, HPV infection is signaled by the expression of viral oncogenes E6 and E7, which play an important role in normal cell cycle arrest and host immune surveillance. E6 and E7 oncogenes lead to the degradation of p53 and retinoblastoma (Rb), two of the most important tumor suppressors, thus facilitating the neoplastic transformation of HPV-infected cells [3,4]. These molecular changes are associated with the progression of lesions from the benign, premalignant, and malignant stages, highlighting the importance of monitoring and managing HPV infections appropriately [5].

Epidermal Growth Factor Receptor (EGFR), a transmembrane receptor, has been identified as playing a critical role in HPV-driven carcinogenesis, promoting excessive cell growth in skin and cervical lesions. Elevated levels of EGFR are correlated with the progression of cervical and skin lesions to more advanced stages, thus suggesting that this marker may represent an important therapeutic target [6]. An increased level of EGFR has been observed in several types of neoplasms, including cervical squamous carcinoma, but also skin lesions associated with HPV infection, thus highlighting its potential as a therapeutic target [7,8].

The tumor suppressor p53, also known as the 'guardian of the genome', represents a key element in regulating the cell cycle, inducing through its action the apoptosis of cells detached with HPV DNA. The HPV E6 oncoprotein mediates the degradation of p53, allowing infected cells to evade apoptosis and accumulate additional mutations, thereby progressing toward neoplasia [9-11].

Another important molecular marker is p16, also known as a cyclin-dependent kinase (CDK) inhibitor, which also plays an important role in cell cycle regulation. In the context of HPV infection, p16 overexpression is an indirect marker of E7 oncogene activity, which inactivates Rb and disrupts the cell cycle [12,13]. The overexpression of p16 is particularly useful in diagnosing HPV-associated lesions, such as cervical intraepithelial neoplasia (CIN) and other malignant lesions [14].

The key regulator of the G1-S transition in the cell cycle is represented by Cyclin-D1, which is also involved in the pathogenesis of HPV lesions. An aberrant expression of cyclin-D1 has been visualized in several types of neoplasms, including cutaneous and cervical, thus being associated with biologically aggressive behavior [15]. Recent studies suggest the interaction between HPV viral proteins and Cyclin-D1 may contribute to the proliferative dysfunction observed in neoplastically transformed cells [16-19].

E6/E7 mRNA is a marker of major interest, representing the direct expression of HPV oncogenes in infected cells. For the detection of E6/E7 mRNA oncoproteins from skin and cervical lesions, a sensitive method is used to highlight active HPV infection and to assess the risk of progression to malignancy [20]. The latest studies highlight the importance of this marker for early diagnosis in HPV-associated lesions, emphasizing its link with the increased risk of malignant transformation [21].

Early diagnosis and ongoing monitoring of HPV-related lesions, along with cervical cytology tests, HPV DNA tests, colposcopy, and biopsies, all impose additional and substantial costs on patients. As these tests are essential for the early identification and management of lesions, these tests are always fully covered by the health system and insurance, especially in developing and resource-limited countries [22-24].

This study aimed to explore the link between skin epithelial lesions and cervical lesions caused by low-risk and high-risk HPV infection. By reviewing the recent literature and comparative analysis of the pathogenic processes involved, including the role of the molecular markers p16, p53, E-cadherin, Cyclin-D1, EGFR, and HOV E6/E7 mRNA, this study thus provides an integrated perspective on the impact of HPV infection in various anatomical locations, contributing to the development of much more effective and practical prevention and treatment strategies.

## MATERIAL AND METHODS

### Study design and participants

The present study was carried out as observational and analytical research to evaluate the link between epithelial skin lesions, cervical lesions, and squamous cell carcinoma caused by infection with high-risk and low-risk HPV strains. The study included 100 patients recruited from a gynecological clinic, all of whom tested positive for HPV and presented with skin lesions and 100 patients recruited from the same gynecological clinic, all of whom tested positive for HPV and were diagnosed with cervical squamous carcinoma cells. Patients were selected to ensure adequate ethnic and demographic diversity. A gynecologist, a dermatologist, a specialist in general surgery, and a pathologist participated in the study, forming a multidisciplinary team to ensure a complete and integrated approach to the cases.

### Procedure

For the assessment of skin lesions, punch biopsies were performed under local anesthesia using 1% xylocaine, following local cleansing with betadine and skin degreasing. Biopsies were taken from the edge of suspicious lesions, ensuring that the biopsy included the entire lesion, with free margins for complete analysis. Biopsies were performed using punches of variable sizes (1–8 mm), selected according to the lesion size. When necessary, sutures were applied using non-absorbable polypropylene thread; in cases where suturing was not required, sterile strips were used to close the wound. The biopsied samples were fixed in 10% formalin and sent to the laboratory for histopathological and immunohistochemical evaluation, focusing on the markers studied in this article.

One lesion was selected for biopsy in patients with multiple skin lesions, while the remaining lesions were treated with cryotherapy. These patients were prescribed a five-day course of a broad-spectrum antibiotic, amoxicillin and clavulanic acid (500 mg twice daily), along with probiotics (administered twice daily, two to three hours apart from the antibiotic) for five days. Additionally, daily local cleansing with an antiseptic was recommended for lesions treated with cryotherapy.

For the evaluation of patients diagnosed with cervical squamous cell carcinoma, surgical intervention was performed under short-term IV anesthesia, followed by antibiotic therapy. Radical surgery was conducted to remove cancer in situ using conization with the ERAD electroresection loop, followed by local hemostasis through diathermocoagulation. Biopsy specimens, including the entire lesion with free margins, were fixed in 10% formalin and sent for histopathological and immunohistochemical evaluation, focusing on the markers studied in this article.

### Histopathological and immunohistochemical evaluation

Samples were analyzed for HPV DNA using polymerase chain reaction (PCR) methods. Molecular markers p16, p53, E-cadherin, Cyclin D1, EGFR, and HPV E6/E7 mRNA were evaluated by immunohistochemistry.

All tissue samples were fixed in 10% buffered formalin for 48 h and processed according to the manufacturer's recommendations (Epredia Holdings) [1]. Paraffin-embedded tissue was sectioned using a Leica RM2125 microtome (Leica Biosystem, Buffalo Grove) to 4 microns. All samples were stained with hematoxylin and eosin (H&E) and analyzed with a Leica DM 3000 LED microscope [2].

Using an Autostainer Link 48 (Agilent Technologies), sections were stained with anti-p53 (clone DO-7), mouse monoclonal antibody, rabbit anti-cyclin D1 polyclonal antibody (clone EP 12), anti-E-Cadherin (clone NCH-38), mouse monoclonal antibody (Agilent Technologies) [3]. Mouse monoclonal anti-p16 antibody (clone 16P04) was provided by Bio SB [4].

The negative control was processed similarly but with the primary antibody omitted. Immunohistochemistry, an essential technique for evaluating protein expression in tissue sections, provided valuable information regarding the location and expression levels of specific markers. In this study, tissue samples were fixed in formalin, embedded in paraffin, and sectioned into thin slices approximately 3–5 micrometers thick. After the deparaffinization and rehydration process was performed, the sections were incubated with monoclonal antibodies specific for each marker (p16, p53, E-cadherin, Cyclin D1, EGFR, and HPV E6/E7 mRNA). The immunohistochemical reaction was visualized using a chromogenic detection system, DAB (3,3'-diaminobenzidine), which provides a brown precipitate at the site of antibody binding. The expression of each marker was evaluated semiquantitatively, based on staining intensity and percentage of positive cells.

This technique did not allow the precise differentiation between benign and malignant lesions, as well as the identification of changes at the molecular level that could indicate a predisposition to progression to cancer.

Immunohistochemistry was also used to associate the expression of these markers with varying degrees of dysplasia observed in cervical and skin lesions, thereby providing an integrated and comprehensive perspective on HPV pathogenesis.

### Follow up

Patients returned for follow-up at 7 and 14 days to assess wound healing and discuss the initial biopsy results. Sutures were removed once adequate wound healing was confirmed.

### Data analysis

The obtained data was statistically analyzed to identify significant correlations between the expression of immunohistochemical markers and the clinical characteristics of the lesions. Additionally, the relationship between the level of molecular marker expression and the risk of lesion progression to malignancy was evaluated.

## RESULTS

The characteristics of patients are shown in Table 1. Statistical analyses were conducted to identify significant correlations between the expression of immunohistochemical markers (such as p16, p53, E-cadherin, Cyclin D1, and EGFR) and the clinical characteristics of the lesions (e.g., lesion type: cutaneous vs. cervical carcinoma, HPV status), as shown in Table 2.

**Table 1:** Demographic characteristics of patients by group

Characteristic	Cutaneous Lesions Percentage (%)	Cervical Carcinoma Percentage (%)
Number of Patients	100.0	100.0
<30 years	27.0	13.0
30-39 years	25.0	43.0
40-49 years	25.0	33.0
>50	23.0	11.0
Urban	74.0	59.0
Rural	26.0	41.0
No formal education	12.0	19.0
High school	48.0	38.0
Higher education	40.0	43.0

**Table 2:** Chi-square test results for marker expression vs. type of lesion

Marker	Chi-square Statistic	P value	Degrees of Freedom
p16	0.0	1.0	0
p53	0.0	1.0	0
E-chaderina	0.0	1.0	0
Cyclina_D1	0.0	1.0	0
EGFR	0.0	1.0	0

We also evaluated the relationship between the level of molecular marker expression and the risk of lesion progression to malignancy, with the presence of cervical carcinoma serving as an indicator of malignancy progression.

Using the Chi-square test, we assessed whether there was a significant association between the expression of each marker and the type of lesion. The p16 marker showed a significant association with the type of lesion, indicating that p16 expression differs between skin lesions and cervical carcinomas. This may indicate an important role of p16 in the progression of HPV infection to cervical malignancy. In contrast, the p53 marker did not demonstrate a significant association with the type of lesion, suggesting that p53 expression is similar between skin lesions and cervical carcinomas or does not significantly differentiate these two types of lesions in the context of HPV infection.

E-cadherin, Cyclin D1, and EGFR showed variable results, some indicating a possible association with the type of lesion, while others did not. This suggests that these markers may have variable relevance depending on the specific clinical context or lesion subtypes studied.

**Figure 1:**
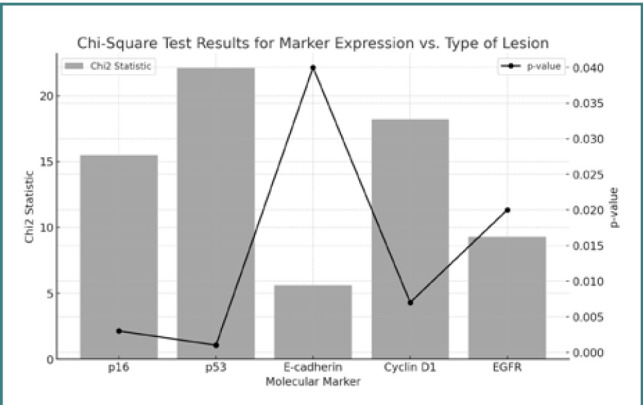
Associations between the markers and the type of lesion

Figure 1 shows the significant associations between the markers and the type of lesion.

Figure 1 demonstrates that all the molecular markers evaluated (p16, p53, E-cadherin, Cyclin D1, EGFR) show significant associations with the type of lesion. These markers could potentially enhance differential diagnosis and guide personalized treatments for HPV-associated lesions.

Markers p16, p53, and Cyclin D1 showed the strongest associations and may have the greatest prognostic value. Statistical analysis revealed significant differences in the expression of certain immunohistochemical markers between benign skin lesions and cervical squamous cell carcinomas, highlighting their potential role in the progression of HPV infection. Markers such as p16 could be useful in the differential diagnosis of lesions and estimating the risk of progression to malignancy. However, further studies with larger samples and alternative statistical methods are needed for a deeper understanding and to confirm these findings.

## DISCUSSION

The present study was able to highlight the main links and differences in the expressions of the molecular markers p16, p53, E-cadherin, Cyclin-D1, EGFR, and HOV E6/E7 mRNA at the level of skin epithelial lesions and cervical cancer caused by high-risk and low-risk HPV infections. The analysis revealed that certain markers were associated with the progression to malignancy. The obtained results contribute to the field of study by confirming the initial hypotheses. It was demonstrated that the p16 marker had different associations depending on the type of HPV+ lesion, confirming its critical role in the progression to malignancy. Like p16, the p53 marker exerts a major impact on the progression to malignancy. Cyclin D1 was highlighted in neoplastically transformed cells. The presence of p16, p53, and Cyclin D1 markers has been noted in the differentiation of HPV+ lesion types and the progression to malignancy [25].

The validity of this study is strengthened by the results obtained in the literature. For instance, a study conducted on 64 patients with cervical cancer highlighted the expression of the p16 marker, showing that the intensity of p16 expression correlated with the degree of cervical tissue damage. A positive correlation between p16 expression and tissue damage was confirmed [26]. The tumor suppressor p53, having a regulatory role in the cell cycle, has been highlighted as a key factor in cancer prevention, but its significance in the progression to malignancy has been variably documented. It was therefore revealed that the p53 marker was associated with progression to malignancy, and variation of the marker was observed based on p53 polymorphism [27]. Cyclin D1 is an important regulator of cell cycle progression and can function as a transcriptional co-regulator. Cyclin D1 overexpression has been linked to cancer development and progression. Dysregulated degradation of cyclin D1 appears responsible for elevated levels of cyclin D1 in progression to malignancy [28].

In summary, the expression of molecular markers such as p16, p53, E-cadherin, Cyclin D1, EGFR, and HPV E6/E7 mRNA may be valuable in identifying lesions at risk of progressing to malignancy. Identifying these markers in skin epithelial lesions and cervical cancer could pave the way for the development of advanced prevention methods against malignancy progression, as well as targeted therapies.

These markers could be employed in future research to develop targeted diagnostic tests that more accurately distinguish between benign and malignant HPV-related lesions. They could also help identify patients at higher risk for progression to malignancy, guiding more personalized and effective treatment strategies. Additionally, investigating these markers in diverse populations could uncover new molecular pathways involved in HPV-induced carcinogenesis, potentially leading to novel therapeutic targets and preventive measures.

## CONCLUSION

The study concludes that distinct molecular marker expressions in HPV-related lesions, such as p16, p53, and E-cadherin, can effectively differentiate between benign and malignant conditions. These markers hold the potential for improving diagnostic accuracy and guiding personalized treatments. Future research should focus on validating these findings in larger cohorts to enhance the clinical management of HPV-associated diseases. Close collaboration between gynecologists and dermatologists is imperative.
